# Differentiating Borderline from Malignant Ovarian-Adnexal Tumours: A Multimodal Predictive Approach Joining Clinical, Analytic, and MRI Parameters

**DOI:** 10.3390/cancers18030516

**Published:** 2026-02-04

**Authors:** Lledó Cabedo, Carmen Sebastià, Meritxell Munmany, Adela Saco, Eduardo Gallardo, Olatz Sáenz de Argandoña, Gonzalo Peón, Josep Lluís Carrasco, Carlos Nicolau

**Affiliations:** 1 Abdominopelvic Imaging Unit, Department of Radiology, Hospital Clínic de Barcelona, 08036 Barcelona, Spaincnicolau@clinic.cat (C.N.); 2 Faculty of Medicine and Health Sciences, University of Barcelona, 08036 Barcelona, Spain; 3 Institut d’Investigacions Biomèdiques August Pi i Sunyer (IDIBAPS), 08036 Barcelona, Spainmasaco@clinic.cat (A.S.); 4 Gynecologic Oncology Unit, Department of Obstetrics and Gynecology, Hospital Clínic de Barcelona, 08036 Barcelona, Spain; 5 Gynecologic Pathology Unit, Department of Pathology, Hospital Clínic de Barcelona, 08036 Barcelona, Spain; 6 Radiology Deparment, Hospital Dr. Sotero Del Rio, Av. Concha y Toro 3459, Puente Alto 8150215, Chile; eduardo.gallardo@falp.org

**Keywords:** O-RADS MRI, borderline ovarian-adnexal tumour (BOT), ovarian-adnexal mass, diffusion-weighted imaging (DWI), decision-tree model, ovarian cancer

## Abstract

**Objectives:** To improve the differentiation of borderline ovarian-adnexal tumours (BOTs) from malignant ovarian-adnexal masses, most of which fall into the indeterminate O-RADS MRI 4 category, by developing a multimodal predictive model that integrates clinical, analytic, and MRI parameters. **Methods:** This retrospective, single-centre study included 248 women who underwent standardised MRI for ovarian-adnexal mass characterisation between 2019 and 2024. Of these, 201 had true ovarian-adnexal masses (114 benign, 22 borderline, and 65 malignant), confirmed by histopathology or stability after ≥12-month follow-up. Forty-one clinical, laboratory, and imaging variables were initially assessed, and after a bivariate evaluation, 18 final predictors with clinical relevance were selected for model construction with thresholds learned from the data. A classification and regression tree (CART) model (“Full Model”) was applied as a second-stage tool after O-RADS MRI scoring, using 10-fold cross-validation to prevent overfitting. A pruned “Simplified Model” was also derived to enhance interpretability. **Results:** O-RADS MRI performed well at the extremes (scores 2–3 and 5) but showed limited discrimination between BOTs and malignancies within category 4 (PPV for borderline = 0.50). The decision-tree models significantly improved diagnostic performance, increasing overall accuracy from 0.856 with O-RADS MRI alone to 0.905 (Simplified Model) and 0.955 (Full Model). The PPV for BOTs within the intermediate O-RADS MRI 4 category increased from 0.49 with O-RADS MRI alone to 0.77 and 0.90 with the simplified and full models, respectively, while maintaining high accuracy for benign and malignant lesions. **Conclusions:** In this retrospective single-centre cohort, the addition of an interpretable rule-based predictive model as a second-line tool within O-RADS MRI category 4 was associated with improved discrimination between borderline and invasive malignant ovarian-adnexal tumours. These findings suggest that multimodal integration of clinical, laboratory, and MRI features may help refine risk stratification in indeterminate cases; however, external validation in prospective multicentre cohorts is required before clinical implementation.

## Simple Summary

Borderline ovarian-adnexal tumours (BOTs) have a much better prognosis than invasive ovarian cancer but are frequently misclassified as malignant by MRI examination applying the “Ovarian-Adnexal Reporting Data System for Magnetic Resonance Imaging (O-RADS MRI)”, especially in score 4. This sometimes leads to overtreatment and potential loss of fertility. In this retrospective single-centre study, we explored whether combining clinical information, blood tumour markers, and MRI features could improve this distinction in indeterminate cases. Our multimodal, simple, rule-based predictive model—used as a second step after O-RADS MRI—significantly improves the diagnostic performance for BOTs. This approach could optimise patient management and directly address a major limitation of current O-RADS MRI classification. Further validation in larger, multicentre studies is required before routine clinical use.

## 1. Introduction

Ovarian-adnexal masses are common in gynaecological practice, covering a wide spectrum from benign physiological findings to aggressive malignancies [1,2].

Although ultrasound (US) is the initial imaging modality for assessing ovarian-adnexal lesions, up to 31% of these masses remain indeterminate or unclassifiable after initial US examination [3]. Moreover, benign solid ovarian-adnexal tumours, such as fibromas, may be misclassified by US in up to 32% of cases [4,5]. Magnetic Resonance Imaging (MRI) has, therefore, become the preferred problem-solving tool for accurate lesion characterisation [6,7,8,9]. The American College of Radiology (ACR) developed the Ovarian-Adnexal Reporting and Data System for MRI (O-RADS MRI) to standardise risk stratification [10,11]. This system was designed for high specificity and positive predictive value (PPV), demonstrating over 90% sensitivity and specificity for malignancy detection [11,12].

However, a major limitation of the O-RADS MRI system lies in category 4 (intermediate-risk), considered the “Achilles heel” of this classification, where malignancy prevalence varies widely from 5% to 90% across different studies [13,14]. This category frequently includes both borderline ovarian tumours (BOTs) and invasive malignant tumours, entities that differ substantially in prognosis and surgical management [6,15,16]. As a result, O-RADS MRI category 4 represents a clinically challenging subgroup in which preoperative decision-making remains difficult [17,18,19].

BOTs are a subtype of epithelial ovarian-adnexal neoplasms representing approximately 10–20% of all epithelial ovarian-adnexal tumours, characterised histologically by epithelial proliferation and nuclear atypia without destructive stromal invasion, and have a significantly better prognosis than invasive cancers [15,16,20,21]. The biological behaviour of BOTs lies on an intermediate spectrum between benign and malignant neoplasms [16,22]. BOTs are characterised by a significantly favourable prognosis due to the absence of stromal invasion, while maintaining a low but persistent risk of recurrence (10–20% after surgery) and a potential for malignant transformation into invasive carcinoma over the long term, occurring in approximately 20% of recurrences [20].

These BOTs predominantly affect younger women of reproductive age, with nearly one-third of patients diagnosed before the age of 40 years, making the balance between oncologic safety and fertility preservation a central issue in clinical management [20]. Surgical management of BOTs typically involves conservative, fertility-sparing procedures like tumourectomy or unilateral salpingo-oophorectomy, contrasting with extensive cytoreductive surgery usually chosen for malignant ovarian-adnexal tumours [21,23,24]. Consequently, an accurate preoperative diagnosis is essential to optimise patient management, avoiding overtreatment of benign lesions and ensuring timely referral of malignant cases to specialised oncology centres [24,25,26,27,28].

Recent research has focused on refining the differentiation of BOTs from invasive carcinomas using advanced MRI techniques and integrated diagnostic approaches. Studies analysing diffusion-weighted imaging (DWI) and apparent diffusion coefficient (ADC) values, such as those by Bourourou et al. (2024) and Hottat et al. [14,26], have demonstrated that specific ADC thresholds can improve risk stratification within the intermediate O-RADS MRI 4 category [14,26]. Beyond imaging, integrative approaches combining MRI with biomarkers, like the study by Xu et al., have shown potential to improve diagnostic performance for BOTs [20].

Despite the growing interest in using quantitative MRI parameters and biomarkers to refine ovarian-adnexal mass characterisation, further research is needed to enhance the clinical applicability and interpretability of predictive models in ovarian-adnexal tumour characterisation. Our study aims to develop an interpretable, rule-based, multimodal predictive model that integrates clinical, analytical, and MRI-derived parameters to refine stratification within O-RADS MRI 4 category. We hypothesised that combining routinely available clinical information and quantitative MRI parameters as the second step after O-RADS MRI assessment could improve discrimination between borderline ovarian tumours and invasive malignancies in this subgroup compared with O-RADS MRI assessment alone. Unlike prior approaches focusing on single imaging features or black-box models, this strategy emphasises interpretability and integration of multimodal data that are routinely available in clinical practice.

## 2. Materials and Methods

This single-centre study received approval from our institutional review board and was conducted following the principles for medical research involving human subjects, according to the Declaration of Helsinki [27]. Informed consent was waived due to the retrospective design and use of previously acquired clinical and imaging data. This study was conducted in a tertiary referral centre for ovarian cancer, with dedicated gynaecologic sonographers and radiologists experienced in ovarian-adnexal mass characterisation.

### 2.1. Patients

We included all consecutive patients who underwent pelvic MRI between January 2019 and December 2024 for the evaluation of ovarian-adnexal masses at our institution. Eligible patients were those presenting with sonographically indeterminate or solid-hypervascular ovarian-adnexal lesions on transvaginal US performed according to the IOTA Simple Rules (IOTA-SR), modified by expert clinical judgement; these patients subsequently underwent a complete O-RADS MRI protocol, with either histopathological confirmation after surgery or ≥12 months of clinical and imaging follow-up [11].

Patients categorised as O-RADS MRI 1 (absence of an ovarian-adnexal lesion) were excluded from further analysis, as they did not correspond to the target condition of this study. Ovarian-adnexal lesions were classified as benign, borderline, or malignant based on histopathology. Malignant ovarian-adnexal tumours were analysed as a single group, as the primary objective of this study was not histological subtyping of invasive malignancies, but rather preoperative discrimination between borderline and invasive disease within the O-RADS MRI framework, reflecting clinically relevant treatment decision-making. For patients who did not undergo surgery, a benign outcome was assigned when imaging findings remained stable for at least 12 months. Follow-up was primarily performed using gynaecologic ultrasound, in accordance with routine clinical practice. Due to the retrospective design, detailed baseline characteristics and the exact follow-up duration beyond 12 months were not consistently available. Serous and mucinous BOTs were considered as a single category due to their similar clinical management [19]. In patients with multiple ovarian-adnexal lesions, the mass with the highest O-RADS MRI score was selected for analysis.

### 2.2. MRI Acquisition and Analysis

All MRI examinations were performed using different 1.5T or 3T scanners (SIGNA Explorer, GE Healthcare, Barcelona, Spain; MAGNETOM Sola Fit and MAGNETOM Prisma Fit Siemens Healthineers, Barcelona, Spain). The protocol, designed in accordance with previously published guidelines, included all sequences required to apply the O-RADS MRI classification: T1-weighted imaging with and without fat saturation, multiplanar T2-weighted imaging, diffusion-weighted imaging (DWI) performed using a b-value of 1000 s/mm^2^, apparent diffusion coefficient (ADC) map, and dynamic contrast-enhanced (DCE) sequences, including one pre-contrast acquisition followed by serial post-contrast acquisitions every 15 s for a total duration of 3.5 min [11,28,29].

Interpretation was independently performed by three radiologists with different levels of expertise, each reading a separate subset of cases, and blinded to clinical, US, and histopathological information; therefore, formal assessment of inter-reader agreement could not be performed. Lesions were scored using the O-RADS MRI lexicon (scores 1–5) [29,30]. ADC measurements were obtained using a single ROI placed within the visually most hypointense region of the enhancing solid component. The ROI was manually drawn on the axial slice of the ADC map. A single ROI was used per lesion, and the mean ADC value was recorded for analysis. ROI placement was visually guided to maximise inclusion of the most diffusion-restricted solid tissue while avoiding cystic, necrotic, or haemorrhagic areas. No minimum or fixed ROI size was imposed due to lesion heterogeneity. A gadolinium-based contrast agent (Gadovist) was administered intravenously at a standard weight-adjusted dose, followed by a saline flush, using an automatic injector. Dynamic acquisition started immediately after contrast injection, with time zero defined as the first post-contrast acquisition. Signal intensity measurements were obtained from an ROI placed within the enhancing solid component and normalised to a reference ROI in the outer myometrium to calculate the enhancement ratio. Dynamic contrast-enhanced (DCE) data were processed using Syngo.via software, version VB80 (Siemens Healthineers, Erlangen, Germany), and time-intensity curves (TICs) were generated and interpreted by each reader. The following 21 image features were recorded for each ovarian-adnexal mass, according to recommendations from the O-RADS MRI lexicon white paper and consensus with the gynaecologic pathologist team in our institution [29]: number of ovarian-adnexal lesions; laterality; presence of peritoneal carcinomatosis; lesion size (longest axial diameter); signal heterogeneity; lesion type (cystic, solid, or mixed); presence of intralesional fat; presence and number of locules in cystic lesions; presence of enhancing solid tissue; signal intensity of solid components on T1- and T2-weighted sequences; diffusion restriction within the solid tissue; ADC values of both solid and cystic components; type of contrast-enhancement curve (low-, intermediate-, or high-risk pattern); time to peak enhancement; peak enhancement ratio (relative to myometrium); absence or presence of aggressive imaging features (including necrosis, haemorrhage, or ill-defined borders); cystic content (serous and/or mucinous); wall thickness (thin or thick with a cutoff value of 2.5 mm); presence of papillary projections, presence of multiple locules (>5); and the presence of locules with differing signal intensities.

### 2.3. Clinicopathological Data

Clinical records were reviewed to collect demographic and clinical variables, including age, menopausal status, presence of pelvic pain at the time of MRI, oncological personal and family history, serum tumour marker values (CA125 and HE4), and US findings based on the IOTA-SR system modified by expert clinical judgement. Management strategies (surgical intervention or imaging follow-up) and final outcomes—either histopathological diagnosis or radiological stability at one-year follow-up—were documented. All variables were collected for every patient as they formed part of the standard items routinely documented during clinical follow-up.

### 2.4. Statistical Analysis

In our statistical analysis, we first evaluated the diagnostic performance of the O-RADS MRI classification alone. Sensitivity, specificity, positive predictive value (PPV), and negative predictive value (NPV) were calculated separately for benign, borderline, and malignant masses using a one-vs-rest approach. For each tumour category, the corresponding O-RADS MRI category was considered a positive test result, while the remaining categories were considered negative. Then, we performed a bivariate evaluation of 41 clinical, analytical, and imaging variables selected based on the literature and medical consensus for further review [20,21,31,32] in relation to the histopathological outcomes or stability after ≥12 months of follow-up. Categorical variables were compared using Fisher’s exact test or chi-square test, and continuous variables using Wilcoxon’s or Kruskal–Wallis tests, as appropriate. Variables with a *p*-value < 0.20 were considered for model development using a deliberately liberal threshold to avoid premature exclusion of potentially relevant predictors.

While more complex risk prediction models have been proposed in the literature, rule-based and descriptor-based approaches (for example, IOTA Simple Rules and O-RADS MRI) are widely used and are relevant resources for tumour identification [33,34,35]. In our study, the decision model chosen was a classification and regression tree (CART) model (first proposed by Beiman et al. [35]), which was developed as a second-line tool applied after O-RADS MRI scoring for all categories. The model was constructed using the Rpart package to identify rule-based combinations of predictors that could improve diagnostic accuracy (Therneau and Atkinson [36]). Model fitting included a 10-fold cross-validation to reduce overfitting. Tree complexity was controlled using cost–complexity pruning as implemented in the Rpart package. The optimal tree size was selected based on the cross-validated error associated with the complexity parameter (cp). A simplified pruned tree was chosen using the one-standard-error rule, retaining the smallest tree whose cross-validated error was within one standard error of the minimum. This pruned version, referred to as the Simplified Model, represented the minimal expansion from the original O-RADS-based classification while preserving comparable cross-validated performance.

## 3. Results

### 3.1. Patients and Clinicopathological Data

**Figure 1:**
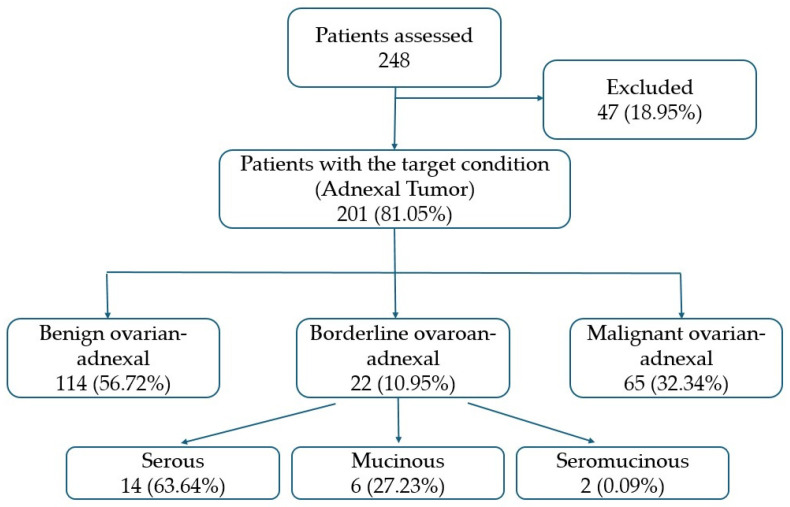
Flow diagram of study participants.

From the 248 patients initially assessed, only 201 presented with ovarian-adnexal masses, all of whom had the index test properly applied, composing the target population. For the 201 patients (mean age 52; standard deviation 11), only the mass with characteristics of greater malignancy was reviewed. Of these, 161 lesions had histopathological confirmation, whereas 40 were presumed benign based on clinical and imaging stability after one year of follow-up. Among all patients, 114 had benign tumours (56.72%), 22 borderline (10.95%), and 65 had malignant tumours (32.34%) (Figure 1). Among the 22 borderline ovarian tumours, 14 were serous, six mucinous, and two seromucinous tumours.

The clinical characteristics of the patients are summarised in Table 1.

### 3.2. O-RADS MRI System Diagnostic Performance

For the study population of 201 patients with ovarian-adnexal masses, the O-RADS MRI system demonstrated a high PPV for benign tumours at scores 2 (benign) and 3 (low-risk), with a PPV of 0.99 for benign tumours. Also, O-RADS score 5 (high-risk) exhibited a strong PPV of 0.85 for malignant tumours (Table 2).

**Figure 2:**
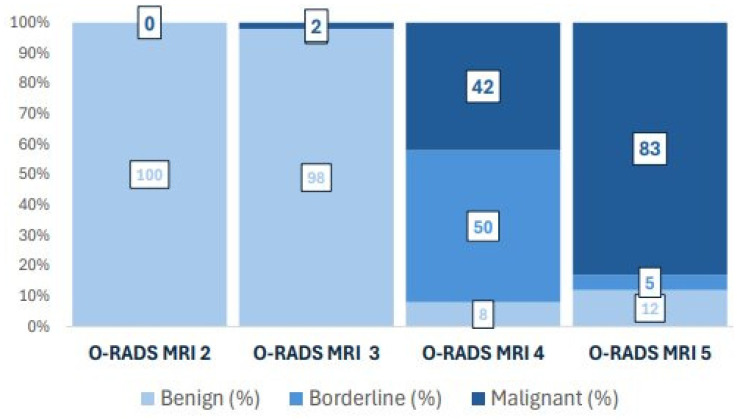
Diagnostic performance of O-RADS MRI categories represented as a bar chart. In score 4, where most borderline tumours are categorised, limited discriminatory ability for malignant tumours is shown.

However, the intermediate-risk category (O-RADS 4) included 50% of BOTs and 42% of malignant tumours (Figure 2). Despite an overall diagnostic accuracy of 0.85, as expected for an indeterminate-risk category, O-RADS MRI category 4 showed limited ability to distinguish borderline tumours from invasive malignancies, resulting in a PPV for BOTs of 0.5 (95% CI: 0.33-0.67) within this subgroup.

### 3.3. Variable Identification and Selection

Bivariate analysis was performed for each of the 41 selected variables on the outcome measure stratified by the index test, through the use of contingency tables and their appropriate test, with particular focus on the O-RADS 4, as it was the category where the index test could not provide a clear separation of the outcome levels (benign, borderline, or malignant). Given the exploratory nature of the analysis, the limited number of borderline tumours, and the objective of selecting a non-zero number of predictors to explore possible rules, a significance level of α = 0.2 was selected to avoid excluding potentially informative predictors.

Following this analysis, 21 predictors showed significant results under this criterion. These included thirteen categorical variables (nine of which were binary markers) and eight numerical variables. Two numerical variables (Ca19.9 value and number of masses) and one categorical variable (presence of intralesional fat) were subsequently removed due to strong concordance with other already-selected markers, refining the set of predictors.

The final set of 18 candidate predictors, which were also checked for their meaningfulness and used in the development of the decision-tree model, included: age; presence of symptoms (pain or bloating at the diagnosis); global US evaluation based on IOTA SR; presence of high tumour markers; HE4 value; Ca125 value; presence of solid tissue; diffusion restriction within the solid tissue; ADC value of solid component; absence of aggressive imaging features; signal intensity of solid components on T1- and T2-weighted sequences; time to peak enhancement; peak enhancement ratio; presence of multiple locules; presence of serous content; presence of thick wall (>2.5 mm); presence of thin wall (<2.5 mm).

### 3.4. Decision-Tree Model Development and BOTs Classification

Once the candidate predictors were identified and it was confirmed that the selected predictors had clear clinical relevance, a decision-tree model (Full Model) and its pruned version (Simplified Model) for classification were fitted. The decision-tree models were evaluated as a second-stage tool applied after O-RADS MRI scoring, with the primary objective of refining discrimination between borderline and invasive malignant tumours within the indeterminate O-RADS MRI category 4. The performance of the Full Model and the Simplified Model demonstrated notable improvements in accuracy over the original O-RADS MRI classification [0.85 (95% CI: 0.799, 0.901) vs. 0.9 (95% CI: 0.856, 0.942) and 0.95 (95% CI: 0.917, 0.979)], even if the only significant difference in classification was against the Full Model. Regarding the different tumours (benign, BOT, or malignant), a major improvement in the PPV was seen for BOTs, from the initial 0.5 (95% CI: 0.329, 0.671) to 0.8 (95% CI: 0.563, 0.943) and 0.9 (95% CI: 0.683, 0.988), respectively. For benign tumours, the PPV remained consistently high across all models, with O-RADS at 0.99 (95% CI: 0.9481, 1); the Simplified Model at 0.99 (95% CI: 0.9481, 1), and the Full Model at 0.96 (95% CI: 0.9141, 0.991). For malignant tumours, the PPV also showed improvement, moving from 0.8333 (95% CI: 0.715, 0.917) with O-RADS to 0.82 (95% CI: 0.71, 0.895) with the Simplified Model and 0.95 with the Full Model (95% CI: 0.871, 0.99) (Table 3). These results indicate that the added value of the decision-tree models is concentrated in the indeterminate O-RADS MRI 4 subgroup, whereas classification at the extremes of the O-RADS spectrum is less changed.

The resulting ruleset and cutoff values for each variable of the Full Model and Simplified Model are shown in Table 4 and Table 5, respectively.

## 4. Discussion

Our study demonstrates that integrating clinical, analytical, and radiological features after O-RADS MRI evaluation increases the accuracy of the O-RADS MRI, particularly in differentiating BOTs from malignant tumours within O-RADS MRI score 4, where these tumours frequently overlap. The O-RADS MRI system performed well in identifying benign lesions at scores 2–3 (PPV = 0.99) and malignant lesions at score 5 (PPV = 0.85). However, its main limitation was the intermediate-risk category (O-RADS 4), where BOTs and invasive malignant tumours were frequently grouped together, resulting in a PPV for BOTs of only 0.50. O-RADS MRI is not intended to identify borderline ovarian tumours as a distinct biological entity, but rather to stratify the risk of malignancy [37]. The present work, therefore, does not seek to replace O-RADS MRI but to complement it by providing additional stratification within the indeterminate O-RADS MRI 4 category, where management decisions are particularly complex. Accordingly, invasive malignant tumours were intentionally grouped as a single outcome category, as clinical management decisions primarily depend on distinguishing borderline disease from invasive malignancy rather than on specific malignant histotypes.

**Figure 3:**
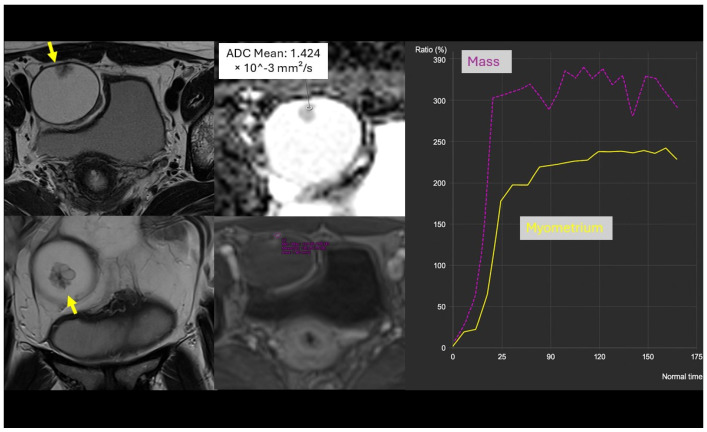
A 32-year-old woman with a right ovarian-adnexal solid-cystic lesion showing a papillary solid component (arrow). The solid component had an ADC value of 1.424 × 10^−3^ mm^2^/s and demonstrated a high-risk time-intensity curve, leading to an O-RADS MRI 5 classification (high risk of malignancy). The patient’s serum HE4 level was 28.8 U/mL. Based on these parameters, the risk of malignancy was recalculated by our full predictive model as a potential borderline tumour. The definitive histopathological diagnosis after surgery confirmed a serous borderline tumour.

**Figure 4:**
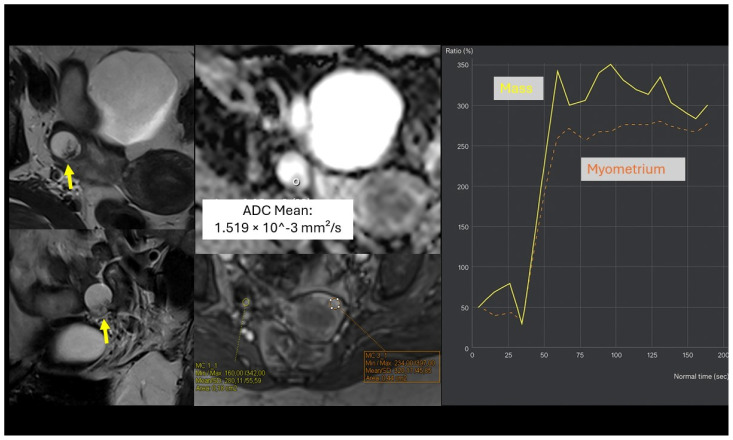
A 52-year-old woman with a right ovarian-adnexal solid-cystic lesion containing a papillary solid component (arrow). The solid component demonstrated an ADC value of 1.519 × 10^−3^ mm^2^/s and a high-risk time-intensity curve, leading to classification as O-RADS MRI 5 (high risk of malignancy). The patient’s serum HE4 level was 30 U/mL, and the malignancy risk was recalculated by our full predictive model as a potential borderline tumour. The definitive histopathological diagnosis after surgery was serous borderline cystadenoma.

We identified 18 key clinical, laboratory, and MRI parameters and integrated them into a CART decision tree applied after the O-RADS MRI score. To ensure model robustness and avoid overfitting, model fitting incorporated a 10-block random cross-validation strategy. A key advantage of the decision-tree approach is its intrinsic capacity for simplification through pruning, which reduces model complexity without compromising cross-validated performance. This two-step approach improved the overall diagnostic accuracy from 0.85 to 0.91 (Simplified Model) and 0.96 (Full Model) and markedly increased the positive predictive value for BOTs to 0.77 and 0.90, respectively (Figure 3 and Figure 4).

Our study corroborated its high diagnostic value for benign (O-RADS 2 and 3) and overtly malignant (O-RADS 5) tumours. This consistently agrees with prior studies reporting O-RADS MRI sensitivities and specificities often exceeding 90% for overall malignancy prediction [31,34,38]. For instance, a meta-analysis by Rizzo et al. reported a pooled sensitivity and specificity higher than 90% for the O-RADS MRI score [31]. Similarly, Kiliçkap et al.’s meta-analysis found a pooled sensitivity of 93.0% and specificity of 90.4% for O-RADS MRI scores ≥4 in ruling out borderline/malignant lesions [32].

However, our results highlighted a critical limitation of the O-RADS MRI system in distinguishing BOTs from malignant tumours within the intermediate-risk O-RADS 4 category, where the PPV for BOTs was only 0.5. This observation aligns with previous reports by Thomassin-Naggara et al. and Rizzo et al. describing O-RADS 4 as the least informative category due to its wide PPV range (5–90%) and high rate of misclassification errors [13,31,39,40]. Wong et al. also noted the marked variability in malignancy prevalence across O-RADS 4 lesions [13]. The difficulty in differentiating BOTs from invasive malignancies in this indeterminate group is a consistent challenge across studies, often leading to the inclusion of BOTs within the malignant category for statistical simplification, thereby limiting specific diagnostic insights [13,31,39].

The decision-tree model approach allowed us to discover potential rules, applied after the O-RADS MRI in a two-stage approach, that might cover the standard pitfalls, particularly addressing its limitations in the O-RADS 4 category [41]. This comprehensive approach aligns with the growing consensus that a multi-parametric assessment is crucial for accurate risk stratification of ovarian-adnexal masses [29,31,34].

Regarding quantitative imaging features, our models included quantitative DWI parameters (ADC value in solid tissue), which proved to be a significant predictor. Although the role of ADC in ovarian-adnexal mass characterisation remains debated due to overlapping values among malignant and some benign histologies (such as endometriomas or cystic teratomas), our results—consistent with recent evidence—support its utility in differentiating BOTs from invasive malignancies [14,26]. Bourourou et al. reported higher ADC values in the solid component of BOTs and proposed a 1.080 × 10^−3^ mm^2^/s cutoff, which is like our optimal threshold (0.993 × 10^−3^ mm^2^/s) that effectively refined BOT classification [14]. Complementary findings by Saida et al. and Ozyilmaz et al. further support the diagnostic relevance of ADC and justify the inclusion of T1- and T2-weighted signal intensity features in our model [42,43]. Higher ADC values within solid components are consistent with the lower cellular density typically observed in borderline tumours compared with invasive carcinomas. Similarly, the absence of aggressive imaging features and lower tumour marker levels reflect the more indolent biological behaviour of borderline lesions.

**Figure 5:**
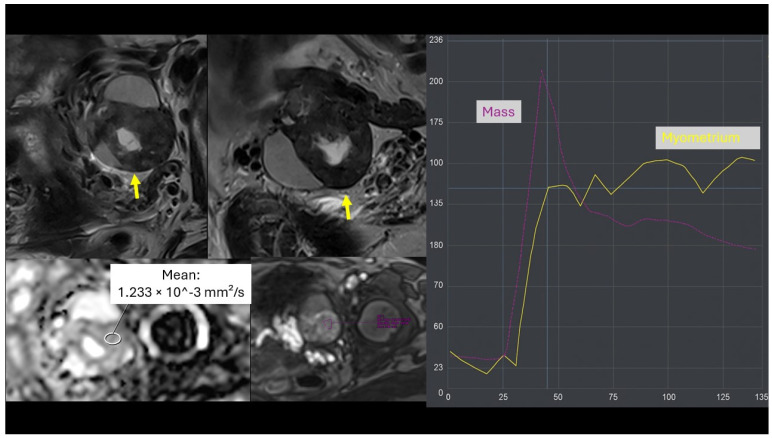
A 64-year-old woman with a right ovarian-adnexal solid-cystic lesion containing a solid component (arrow) and demonstrating a high-risk time-intensity curve, initially classified as O-RADS MRI 5 (high risk of malignancy). The solid portion of the tumour had an ADC value of 1.233 × 10^−3^ mm^2^/s, and the patient’s serum HE4 level was 2 U/mL. Based on these parameters, the malignancy risk was recalculated by our full predictive model as a likely benign tumour. The definitive histopathological diagnosis after surgery was struma ovarii.

In addition, we explored quantitative contrast-enhancement metrics—time to peak and peak enhancement ratio—to objectively assess vascularisation, addressing the limited use of qualitative DCE assessment using TICs in prior studies [28,29]. This approach may improve characterisation of atypical benign lesions with intense enhancement, such as struma ovarii, which can mimic malignancy, and further investigation must be carried out (Figure 5) [44]. While these findings are consistent with previous studies highlighting the value of quantitative MRI parameters, most published approaches either pooled borderline tumours with malignant lesions or relied on complex models with limited interpretability, which may hinder clinical translation [45].

Regarding morphological features, we incorporated key morphological features—such as multilocularity, wall thickness, serous content, solid tissue, and aggressive imaging signs—which are well-established indicators of malignancy risk [13,29,46]. Ozyilmaz et al. reported that mural nodules, the number of locules, and septa or wall thickness (>2.45 mm) helped distinguish benign from borderline and malignant mucinous tumours [43]. Internal branching within papillary structures was more often observed in benign and borderline seromucinous neoplasms, supporting the diagnostic relevance of these features in our variable selection [29].

Regarding clinical and laboratory features, the diagnostic value of tumour markers like CA125 and HE4 is widely recognised in ovarian-adnexal cancer detection and risk algorithms, although their sensitivity and specificity are limited in the early stages [44,46]. Wong et al. proposed combining imaging features with elevated CA-125 for refining risk stratification within the O-RADS MRI 4 category, but did not establish a reproducible model usable in clinical practice [13]. Patient age and reproductive history (nulliparity) are also established risk factors for ovarian-adnexal malignancy [13,20]. Integrating these clinical and biochemical variables with MRI features in a more comprehensive and accurate diagnostic model opens the door to potential clinical application as a complementary tool to standard O-RADS MRI assessment.

Our main contribution lies in the improvement in PPV and specificity in diagnosing BOTs, mainly with the Full Model. Both models enhanced overall diagnostic performance while maintaining high sensitivity, significantly improving O-RADS MRI performance in our cohort and in agreement with what is described in the literature [31]. Unlike many previous studies that grouped BOTs with malignant lesions or lacked sufficient sample size for detailed analysis, our approach specifically addressed this problem by combining imaging, clinical, and analytical information that refined BOT diagnosis [31]. This distinction between borderline and invasive tumours, if confirmed in further studies, could have potential direct clinical implications for surgical planning, including fertility-sparing options. Furthermore, the interpretable nature of decision-tree models facilitates their potential translation into clinical practice, in contrast with less-transparent black-box radiomic approaches.

This study has several important limitations. Its retrospective, single-centre design may limit generalisability and introduce selection bias. In addition, a subset of lesions was classified as benign based on imaging stability during follow-up rather than histopathology, which may not fully exclude indolent or low-grade disease and may introduce verification bias. The relatively small number of borderline tumours increases the risk of instability in decision-tree splits and limits the robustness of threshold estimation. Variability in MRI acquisition parameters across scanners could have affected quantitative metrics such as ADC values, as highlighted by other authors [28,29]. Intra-reader repeatability of the ADC and DCE measurements could not be formally assessed, as each examination was interpreted by a single reader and repeat measurements were not available due to the retrospective design. Despite the use of cross-validation to reduce overfitting, training the model on the entire dataset prevented true internal validation, reinforcing the need for external and multicentric validation before clinical implementation. Furthermore, each examination was interpreted by a single reader, precluding assessment of inter-reader agreement, and MRI acquisitions were performed across different scanners, which may affect the generalisability of quantitative thresholds. Finally, the absence of external validation precludes conclusions regarding clinical implementation.

Given the dependence of ADC and DCE metrics on acquisition parameters and scanner characteristics, the thresholds identified in this study should be interpreted as data-driven estimates that may require site-specific recalibration in external cohorts. Future studies should, therefore, focus on validating and recalibrating the model in larger, multi-institutional cohorts to confirm its reproducibility and clinical applicability. Improved preoperative discrimination between borderline tumours and invasive malignancies within O-RADS MRI category 4 may support more individualised surgical planning, particularly in younger patients for whom fertility-sparing approaches are being considered. However, imaging-based models should be interpreted as adjunctive tools, and cannot replace multidisciplinary clinical decision-making.

## 5. Conclusions

Despite these limitations, our study provides a meaningful contribution by specifically enhancing BOT characterisation within the O-RADS MRI framework. The marked improvement in PPV and specificity indicates that integrating morphological, quantitative, and clinical features within an interpretable decision-tree model may improve discrimination between borderline ovarian tumours and invasive malignancies within the indeterminate O-RADS MRI category 4. By acting as a complementary, second-line tool after standard O-RADS MRI assessment, this approach may help refine preoperative risk stratification in selected cases. However, given the retrospective design, limited number of borderline tumours, and lack of external validation, these findings should be considered preliminary. Prospective, multicentre studies are required to validate the proposed model, assess its reproducibility across different settings, and determine its potential role in clinical decision-making.

## Figures and Tables

**Table 1:** Clinical and biochemical characteristics of the study cohort.

	Target Population	Full Cohort
	*N* = 201 *	*N* = 248 *
Number of ovarian-adnexal masses		
1	162 (81%)	162 (65%)
2	36 (18%)	36 (14.5%)
3	0 (0%)	0 (0%)
≥4	3 (1.5%)	3 (0.1%)
Absence of ovarian-adnexal mass	0 (0%)	47 (19%)
Age	52 (39, 63)	52 (39.62)
Menopause	101 (50%)	117 (47%)
Oncologic history	27 (14%)	27 (11%)
Symptoms		
Asymptomatic	172 (86%)	210 (85%)
Abdominal pain	3 (1.5%)	6 (2.4%)
Fever	0 (0%)	1 (0.4%)
Other	26 (13%)	31 (13%)
Elevated tumour markers	65 (36%)	69 (33%)

* Values are n (%); age is expressed as median (Q1,Q3).

**Table 2:** Diagnostic performance of the O-RADS MRI.

O-RADS MRI Category	Benign Ovarian-Adnexal *n* = 114 *	Borderline Ovarian-Adnexal *n* = 22 *	Malignant Ovarian-Adnexal *n* = 65 *
*Benign–Low-risk (Score 2* *–* *3)*	104 (99%)	1 (1.0%)	0 (0%)
*Indeterminate-risk (Score 4)*	3 (8.3%)	18 (50%)	15 (42%)
*High-risk (Score 5)*	7 (12%)	3 (5.0%)	50 (83%)

* Values are n (%).

**Table 3:** Diagnostic performance of O-RADS MRI compared to the full and simplified models.

	Diagnostic Performance	Sensitivity	Specificity	PPV	NPV
**O-RADS MRI**	0.856 (0.799, 0.901)				
*- Benign*		0.912 (0.845, 0.957)	0.989 (0.938, 1)	0.99 (0.9481, 1)	0.896 (0.817, 0.949)
*- Borderline*		0.818 (0.597, 0.948)	0.899 (0.846, 0.939)	0.5 (0.329, 0.671)	0.976 (0.939, 0.993)
*- Malignant*		0.769 (0.648, 0.865)	0.926 (0.869, 0.964)	0.833 (0.715, 0.917)	0.894 (0.831, 0.939)
**Full Model**	0.955 (0.917, 0.979)				
*- Benign*		0.982 (0.938, 0.998)	0.954 (0.886, 0.987)	0.966 (0.9141, 0.991)	0.976 (0.918, 0.997)
*- Borderline*		0.818 (0.597, 0.948)	0.989 (0.96, 0.999)	0.9 (0.683, 0.988)	0.978 (0.944, 0.994)
*- Malignant*		0.954 (0.871, 0.99)	0.978 (0.937, 0.995)	0.954 (0.871, 0.99)	0.978 (0.937, 0.995)
**Simplified Model**	0.905 (0.856, 0.942)				
*- Benign*		0.912 (0.845, 0.957)	0.989 (0.938, 1)	0.99 (0.9481, 1)	0.896 (0.817, 0.949)
*- Borderline*		0.727 (0.498, 0.893)	0.978 (0.944, 0.994)	0.8 (0.563,0.943)	0.967 (0.929, 0.988)
*- Malignant*		0.954 (0.871, 0.99)	0.897 (0.833, 0.943)	0.816 (0.71, 0.895)	0.976 (0.931, 0.995)

Values are expressed as point estimate (95% CI); PPV = positive predictive value; NPV = negative predictive value. The Simplified Model was derived after pruning with a complexity parameter (cp) of 0.028.

**Table 4:** Full Model: decision rules and diagnostic accuracy. Color coding is used to visually distinguish predicted risk categories: green indicates benign, yellow indicates borderline, and red indicates malignant predictions.

O-RADS MRI	Prevalence *	Decision Criteria	Predicted Risk Category	Observed Prevalence	Diagnostic Accuracy
2–3 (99% Benign)	52% [105]	-	BENIGN	52% [105]	99%
4 (50% Borderline, 42% Malignant)	18% [36]	Solid ADC ≥ 993, age < 67, asymptomatic	BORDERLINE	9% [18]	89%
		Rest	MALIGNANT	9% [18]	77.78%
5 (83% Malignant)	30% [60]	Solid ADC ≥ 993, he4 ≤ 33, enhancement ratio ** < 66	BENIGN	1% [3]	66.67%
		Solid ADC ≥ 993, he4 < 19	BENIGN	2% [4]	75%
		Solid ADC < 993, T1w signal intensity < 67	BENIGN	2% [4]	75%
		Solid ADC ≥ 993, he4 in [19,33]	BORDERLINE	1% [1]	100%
		Rest	MALIGNANT	24% [48]	97.92%

%[N]; ADC expressed in ×10^−3^ mm^2^/s * Prevalence according to O-RADS MRI; ** Enhancement ratio of the solid component of the lesion relative to the myometrium.

**Table 5:** Simplified Model: decision rules and diagnostic accuracy. Color coding is used to visually distinguish predicted risk categories: green indicates benign, yellow indicates borderline, and red indicates malignant predictions.

O-RADS MRI	Prevalence *	Decision Criteria	Predicted Risk Category	Observed Prevalence	Diagnostic Accuracy
2–3 (99% Benign)	52% [105]	-	BENIGN	52% [105]	99%
4 (50% Borderline, 42% Malignant)	18% [36]	Solid ADC ≥ 993, age < 67	BORDERLINE	10% [20]	80%
		Rest	MALIGNANT	8% [16]	68.75%
5 (83% Malignant)	30% [60]	-	MALIGNANT	30% [60]	83%

%[N]; ADC expressed in ×10^−3^ mm^2^/s * Prevalence according to O-RADS MRI.

## Data Availability

The data presented in this study are not publicly available due to ethical and privacy restrictions, as they contain sensitive clinical and imaging information from patients. Anonymized data may be made available from the corresponding authors upon reasonable request and subject to approval by the institutional review board.

## Abbreviations

The following abbreviations are used in this manuscript:

ACR	American College of Radiology
ADC	Apparent diffusion coefficient
AUC	Area Under the Curve
BOT	Borderline ovarian-adnexal tumour
CA125	Cancer Antigen 125
CART	Classification and regression tree
CI	Confidence interval
DCE	Dynamic contrast-enhanced
DWI	Diffusion-weighted imaging
FSS	Fertility-Sparing Surgery
HE4	Human Epididymis Protein 4
ICC	Intraclass Correlation Coefficient
IOTA	International Ovarian Tumour Analysis
MRI	Magnetic Resonance Imaging
NPV	Negative predictive value
O-RADS	Ovarian-Adnexal Reporting and Data System
OR	Odds Ratio
PPV	Positive predictive value
ROI	Region of Interest
SD	Standard deviation
SIR	Signal Intensity Ratio
STUMP	Smooth Muscle Tumour of Uncertain Malignant Potential
TIC	Time-intensity curve
US	Ultrasound
